# Anthocyanins and their physiologically relevant metabolites alter the expression of IL‐6 and VCAM‐1 in CD40L and oxidized LDL challenged vascular endothelial cells

**DOI:** 10.1002/mnfr.201400803

**Published:** 2015-04-30

**Authors:** Hiren P. Amin, Charles Czank, Saki Raheem, Qingzhi Zhang, Nigel P. Botting, Aedín Cassidy, Colin D. Kay

**Affiliations:** ^1^ Department of NutritionNorwich Medical School, University of East AngliaNorwichUK; ^2^ Department of Life Sciences, Faculty of Science and TechnologyUniversity of WestminsterLondonUK; ^3^ School of Chemistry St. Andrews UniversityFifeScotlandUK; ^4^ Present address: Linear Clinical ResearchNedlandsWestern Australia

**Keywords:** Adhesion molecule, Cytokine, Cyanidin‐3‐glucoside, Inflammation, Metabolites

## Abstract

**Scope:**

In vitro and in vivo studies suggest that dietary anthocyanins modulate cardiovascular disease risk; however, given anthocyanins extensive metabolism, it is likely that their degradation products and conjugated metabolites are responsible for this reported bioactivity.

**Methods and results:**

Human vascular endothelial cells were stimulated with either oxidized LDL (oxLDL) or cluster of differentiation 40 ligand (CD40L) and cotreated with cyanidin‐3‐glucoside and 11 of its recently identified metabolites, at 0.1, 1, and 10 μM concentrations. Protein and gene expression of IL‐6 and VCAM‐1 was quantified by ELISA and RT‐qPCR. In oxLDL‐stimulated cells the parent anthocyanin had no effect on IL‐6 production, whereas numerous anthocyanin metabolites significantly reduced IL‐6 protein levels; phase II conjugates of protocatechuic acid produced the greatest effects (>75% reduction, *p* ≤ 0.05). In CD40L‐stimulated cells the anthocyanin and its phase II metabolites reduced IL‐6 protein production, where protocatechuic acid‐4‐sulfate induced the greatest reduction (>96% reduction, *p* ≤ 0.03). Similarly, the anthocyanin and its metabolites reduced VCAM‐1 protein production, with ferulic acid producing the greatest effect (>65% reduction, *p* ≤ 0.04).

**Conclusion:**

These novel data provide evidence to suggest that anthocyanin metabolites are bioactive at physiologically relevant concentrations and have the potential to modulate cardiovascular disease progression by altering the expression of inflammatory mediators.

**Abbreviations**
CD40: cluster of differentiation 40
CD40L: CD40 ligand
CVD: cardiovascular disease
C3G: cyanidin‐3‐glucoside
HUVEC: human umbilical vein endothelial cells
IVA: isovanillic acid
oxLDL: oxidized LDL
PCA: protocatechuic acid
PGA: phloroglucinaldehyde
TNF‐α: tumor necrosis factor‐alpha
TRAF: TNF receptor‐associated factor
VA: vanillic acid
VCAM‐1: vascular cell adhesion molecule‐1

## 1 Introduction

Habitual intake of anthocyanins has been associated with a reduced incidence of cardiovascular disease (CVD) 1. Studies exploring the mechanism of action behind this activity have primarily focused on vascular reactivity, such as endothelial‐dependent nitric oxide mediated vasodilation and blood pressure regulation 2, 3, 4. Recently researchers have begun to explore anti‐inflammatory activities of anthocyanins as a potential cardioprotective mechanism of action and there is now evidence to suggest that anthocyanins may modulate the production of key inflammatory mediators, including IL‐6 and vascular cell adhesion molecule‐1 (VCAM‐1) 5, 6, 7.

Cell adhesion molecules such as VCAM‐1 play a key role in the progression of atherosclerosis as they are involved in the adhesion of leukocytes to the endothelium during the early stages of atherosclerosis 8, 9. Following leukocyte adhesion, IL‐6 is secreted, which further perpetuates inflammation, with eventual progression to atherosclerosis 10, 11, 12. Anthocyanins, in vivo and in vitro, have been demonstrated to modulate the expression of both IL‐6 and VCAM‐1 13, 14, 15. Studies feeding anthocyanin‐rich bilberry and strawberry beverages to human participants with elevated risk of CVD reported reduced plasma concentrations of IL‐6 and C‐reactive protein 7, 13, 16. This is also supported by animal and in vitro studies that demonstrated that IL‐6 production can be attenuated by anthocyanins 14, 17. In addition, black soybean anthocyanins have been observed to reduce VCAM‐1 production in tumor necrosis factor‐α (TNF‐α)‐challenged human endothelial cells and protect against ischemia‐induced myocardial injury in mice 5, 15. In previous studies the assumption is, parent anthocyanins are responsible for the observed biological activity. However, due to the biological instability and extensive metabolism of anthocyanins 18, we propose that the anti‐inflammatory bioactivity results from the activity of anthocyanin degradation products and metabolites and not the parent anthocyanins directly.

**Figure 1:**
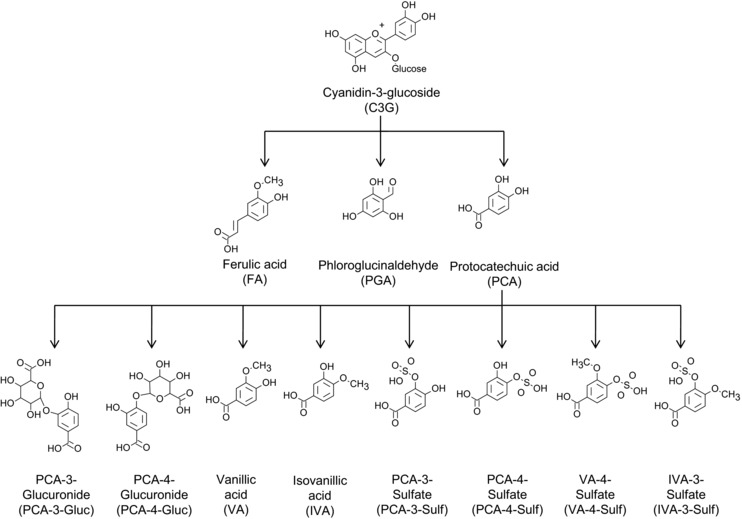
Recently identified metabolites of C3G^1^ screened for their activity against IL‐6 and VCAM‐1 expression in oxLDL‐ and CD40L‐stimulated HUVECs. C3G, cyanidin‐3‐glucoside; PCA, protocatechuic acid, PGA, phloroglucinaldehyde; VA, vanillic acid; IVA, isovanillic acid; PCA‐4‐Gluc, PCA‐4‐glucuronide; PCA‐3‐Gluc, PCA‐3‐glucuronide; PCA‐4‐Sulf, PCA‐4‐sulfate; PCA‐3‐Sulf, PCA‐3‐sulfate; VA‐4‐Sulf, VA‐4‐sulfate; IVA‐3‐Sulf, IVA‐3‐sulfate; FA, ferulic acid. ^1^Metabolites recently identified and reported by de Ferrars 25.

Evidence suggests that after consumption, cyanidin‐3‐glucoside (C3G) degrades to form protocatechuic acid (PCA) and phloroglucinaldehyde (PGA) 18. Previous in vitro studies have shown that phenolic acids, including PCA and the methylated derivative of PCA, vanillic acid (VA), reduce VCAM‐1 production in endothelial cells and IL‐6 production in mouse peritoneal macrophages, respectively 19, 20. Therefore it is likely that these and other metabolites of similar structure contribute towards the anti‐inflammatory properties of anthocyanins. However, presently we have no understanding of the anti‐inflammatory activity of the majority of anthocyanin metabolites, as we have only recently established the identity of the phenolic structures in humans 18. Here we examine the effects of C3G, its degradation products (PCA and PGA) and novel phase II conjugates (methyl, sulfate, glucuronide; Fig. 1) at concentrations observed in vivo (0.1–10 μM) 18, on IL‐6 and VCAM‐1 protein production in human umbilical vein endothelial cells (HUVECs) stimulated with either oxidized LDL (oxLDL) or a cluster of differentiation 40 ligand (CD40L).

## 2 Materials and methods

### 2.1 Materials

HUVEC, large vessel endothelial cell growth medium, growth factors and antibiotic (Amphotericin B/Gentamycin 1000 × concentrated) supplements were obtained from TCS cell works (Buckingham, UK) while RPMI 1640 media, FBS, glutamine and penicillin/streptomycin were from PAA (Kent, UK). D1.1 cells were generously provided by Dr. Maria O'Connell, Department of Pharmacy, University of East Anglia (Norwich, UK). Monoclonal Antibody against cluster of differentiation 40 (CD40) was purchased from Enzo bioscience (Cambridge, UK). LDL was purchased from Millipore (UK). Cyanidin‐3‐glucoside was purchased from Extrasynthese (Genay Cedex, France). Phase II conjugates of phenolic acids (PCA‐3‐glucuronide, PCA‐4‐glucuronide, isovanillic acid‐3‐sulfate, PCA‐4‐sulfate, VA‐4‐sulfate, PCA‐3‐sulfate) were synthesized by St. Andrews University as described previously 21. DuoSet ELISA kits [IL‐6 (DY206) and VCAM‐1 (DY809)], flat bottom clear polystyrene 96 wells ELISA plates (DY990) and reagent diluent (DY995) were purchased from R&D systems Europe, UK. ELISA protein quantification was established using an Omega BMG plate reader (BMG Labtech, Aylesbury, UK). TRIzol^®^ reagent was purchased from Life Technologies (Paisley, UK). RiboLock RNase inhibitor, DNase reaction buffer (with MgCl_2_), DNase I (RNase free), EDTA (50 mM) were purchased from Fisher Scientific (Loughborough, UK). Chloroform (molecular biology grade), isopropanol/propan‐2‐ol and dNTP PCR mix (ready mixed 10 mM) were obtained from Fisher Scientific (Loughborough, UK). PCR Primers and Real Time PCR master mix with SYBR green were supplied by Primer Design (Southhampton, UK), Oligo (dT) primers were purchased from Ambion, Life Technologies (Paisley,UK) and SuperScript^®^ II Reverse Transcriptase, first strand buffer and DTT (100 mM) were from Invitrogen (Paisley, UK). The RT‐qPCR was performed using ABIS7500 system, Life Technologies (Paisley, UK). All other chemicals were from Sigma‐Aldrich (Dorset, UK).

### 2.2 Preparation of cell treatments

C3G, PCA, PGA, VA, isovanillic acid (IVA), PCA‐4‐glucuronide, PCA‐3‐glucuronide, PCA‐4‐sulfate, PCA‐3‐sulfate, vanillic acid‐4‐sulfate, IVA‐3‐sulfate and ferulic acid were assessed at concentrations of 0.1, 1 and 10μM in cell culture media. Standard solutions were initially prepared in 100% DMSO at concentrations of 40 mM for C3G and 25 mM for all other metabolites followed by dilution to final concentrations (0.1, 1 and 10 μM) using cell media, immediately prior to application to cells. Final treatment concentration of DMSO was ≤0.05%.

### 2.3 Cell culture and assays

Early passage, pooled donor HUVECs were used between passages 2–4 and cultured in large vessel endothelial cell growth medium at 37°C and 5% CO_2_. HUVECs were sub‐cultured at ∼90% confluence by trypsinisation and seeded at 60 000 cells/well in 24‐well plates on fibronectin coated surface (0.25 μg/cm^2^) for cell assays. D1.1 cells were maintained in suspension between 6 × 10^5^ cells/mL and 1 × 10^6^ cells/mL in RPMI media at 37°C and 5% CO_2_. For oxLDL‐stimulated experiments, oxLDL was prepared as previously described 22 and 90% confluent HUVECs were co‐incubated with 5 μg/mL oxLDL with or without treatment compounds for 24 h. The oxidation of LDL was confirmed by agarose gel electrophoresis 23. For CD40L‐stimulated assays, 90% confluent HUVECs were co‐incubated with D1.1 cells at 1 × 10^6^ cells/well, with or without treatments for 24 h. The specificity of CD40L stimulation was confirmed by pre‐incubation of D1.1 cells at 1 × 10^6^ cells/well with anti‐CD40L (5 μg/mL) for 1 h followed by their incubation with HUVECs for 24 h. Post 24 h co‐incubation, supernatants and culture plates were stored immediately at –80°C until required for protein and mRNA quantification, except 24‐well plates for the CD40L‐stimulated assay, where the HUVECs were washed three times with 1% warm PBS to remove D1.1 cells before plates were stored at –80°C.

### 2.4 IL‐6 and VCAM‐1 protein quantification by ELISA

Cell supernatants (100 μL) were used to quantify IL‐6 and VCAM‐1 using commercially available DuoSet ELISA kits (according to manufacturer's instructions). Briefly, 96‐well plates were coated with mouse anti‐human IL‐6 and VCAM‐1 primary antibodies followed by incubation for 2 h at room temperature with cell supernatants. Subsequently, plates were incubated with goat anti‐human IL‐6 and VCAM‐1 secondary antibodies for 2 h, followed by the addition of horseradish‐peroxidase conjugated streptavidin HRP, substrate reagents, stabilized H_2_O_2_ and tetramethylbenzidine. The color was developed using 2N H_2_SO_4_ stop solution (50 μL/well) that was measured at 450 nm using a BMG plate reader. The IL‐6 ELISA intra‐ and inter‐assay CV was 4.3 ± 1.3% and 1.49%, respectively (mean ± SD, *n* = 4). The VCAM‐1 intra‐ and inter‐assay CV was 7.6 ± 2.2% and 0.69%, respectively (mean ± SD, *n* = 4).

### 2.5 Quantitative real‐time PCR

RNA extraction from HUVECs was performed using phenol‐chloroform extraction (TRIzol^®^, 500 μL/well). RNA was quantified using a NanoDrop^®^2000 (purity ratio A_260/280_). One micro‐gram of RNA was prepared for reverse transcription by incubations with RiboLock (0.25 μL/reaction), DNase buffer (1 μL/reaction) and DNase (1 μL/reaction) at 37°C for 30 min, followed by incubation with EDTA (50 mM, 1 μL/reaction), oligo (dT) primers (1 μL/reaction) and dNTP PCR mix (1 μL/reaction) at 65°C for 10 min followed by first strand buffer (4 μL/reaction), RiboLock (1 μL/reaction) and DTT (100 mM, 1 μL/reaction) for 2 min at 42°C. Reverse transcription was carried out using SuperScript^®^ II (1 μL/reaction) at 42°C for 50 min followed by inactivation for 15 min at 70°C. Five micro‐liters of 1:10 diluted cDNA solutions (25 ng) was added to plates containing IL‐6 and VCAM‐1 primers (1 μL/reaction), real‐time PCR master mix with SYBR^®^ green (8.33 μL/reaction) and nuclease‐free water (5.67 μL/reaction). The RT‐qPCR was performed using an ABIS7500 where the enzymes were activated at 95°C for 10 min followed by 50 cycles of denaturation (15 s/cycle at 95°C) and data collection (1 min/cycle at 60°C). Target genes, were normalized against two geNorm housekeeping reference genes, UBE2D2 and PRDM4 as established using qbase^PLUS2^ [Biogazelle (Zwinjaarde, Belgium)]. The fold expression of the target genes were quantified by 2^ΔCt^ determination, where ΔCt = Ct _target gene_/Ct _mean Ct of references gene_. Forward and reverse primer sequence for IL‐6 was GCA GAA AAC AAC CTG AAC CTT and ACC TCA AAC TCC AAA AGA CCA and CAG GCT AAG TTA CAT ATT GAT GAC AT and GAG GAA GGG CTG ACC AAG AC for VCAM‐1.

### 2.6 Statistical analysis

Analysis of variance with Tukey post‐hoc tests were performed using SPSS software (IBM, New York, USA) version 18 for Windows. Significance was determined at the level of 5%. Three biological replicates for each of the controls and treatments (plated in technical duplicates) were used for analysis unless otherwise stated, and means of biological replicates were represented graphically. Error bars in figures represent SD.

## 3 Results

### 3.1 OxLDL‐stimulated IL‐6 and VCAM‐1 production

**Figure 2:**
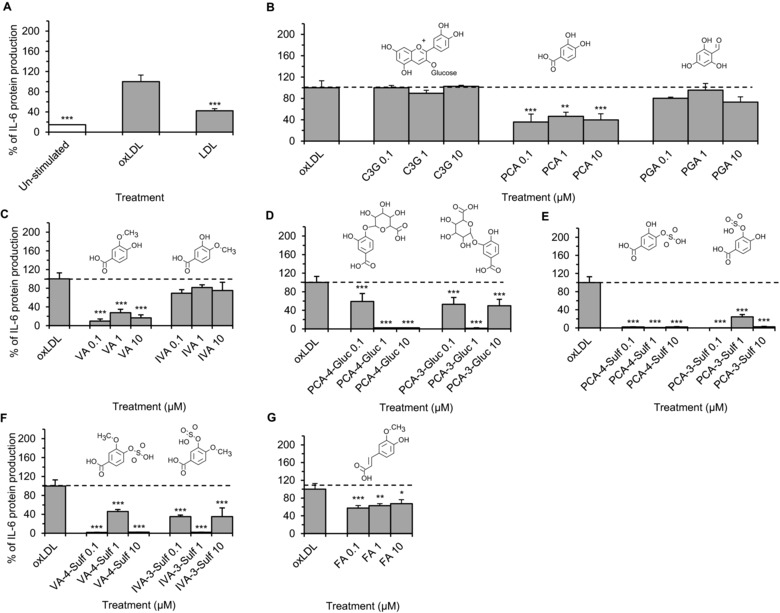
OxLDL‐stimulated IL‐6 production in HUVECs co‐incubated with C3G, phenolic metabolites or LDL controls for 24 h. (A) cells incubated with or without LDL (5 μg/mL); (B–G) cells incubated with C3G or phenolic metabolites at 0.1, 1 and 10 μM and oxLDL. All data expressed as mean percentage (± SD, *n* = 3) of oxLDL‐induced controls (expressed as 100%). ***p ≤ 0.001, **p ≤ 0.01, *p ≤ 0.05 (analysis of variance (ANOVA) with Tukey post‐hoc) relative to oxLDL‐stimulated control cells. C3G, cyanidin‐3‐glucoside; PCA, protocatechuic acid; PGA, phloroglucinaldehyde; VA, vanillic acid; IVA, isovanillic acid; PCA‐4‐Gluc, PCA‐4‐glucuronide; PCA‐3‐Gluc, PCA‐3‐glucuronide; PCA‐3‐Sulf, PCA‐3‐sulfate; PCA‐4‐Sulf, PCA‐4‐sulfate; VA‐4‐Sulf, VA‐4‐sulfate; IVA‐3‐Sulf, IVA‐3‐sulfate; FA, ferulic acid.

OxLDL‐stimulation resulted in a 6.9 ± 0.4‐fold increase in IL‐6 protein relative to the un‐stimulated control (*p* ≤ 0.001, Fig. 2A); whereas there was no effect of oxLDL on VCAM‐1 protein production (data not shown). There was no effect of the parent anthocyanin under oxLDL stimulated conditions, while nine metabolites reduced oxLDL‐induced IL‐6 production by >32% (*p* ≤ 0.05, Fig. 2) relative to the cells treated with oxLDL alone (oxLDL control), at one or more of the tested treatment concentrations (0.1, 1, 10 μM). PCA, reduced IL‐6 production by ≥53.6 ± 7.6% (*p* ≤ 0.001, Fig. 2B) while PGA had no effect. Among the methyl, glucuronide and sulfate conjugated metabolites of PCA, PCA glucuronides and PCA sulfates elicited the greatest reductions in IL‐6 production (across all concentrations tested), with maximum reductions (99.1 ± 0.1%, *p* ≤ 0.001) observed for the sulfate conjugates (Fig. 2C–E). In addition, VA‐4‐sulfate, IVA‐3‐sulfate and ferulic acid also displayed bioactivity against oxLDL‐induced IL‐6 production (between 54.1 ± 4.4 and 98.2 ± 0.2% reduction, *p* ≤ 0.05; Fig. 2F and G).

### 3.2 OxLDL‐stimulated IL‐6 mRNA levels

**Figure 3:**
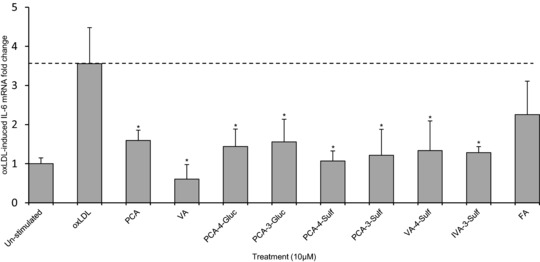
Change in OxLDL‐stimulated IL‐6 mRNA in HUVECs co‐incubated with C3G and phenolic metabolites at 10 μM or oxLDL controls for 24 h. All graphical data expressed as mean fold change (± SD, *n* = 3) of un‐stimulated (basal) IL‐6 mRNA levels. *p ≤ 0.05 (ANOVA with Tukey post‐hoc) relative to oxLDL‐stimulated control. PCA, protocatechuic acid; VA, vanillic acid; PCA‐3‐Sulf, PCA‐3‐sulfate; PCA‐4‐Sulf, PCA‐4‐sulfate; VA‐4‐Sulf, VA‐4‐sulfate; IVA‐3‐Sulf, IVA‐3‐sulfate; FA, ferulic acid.

The compounds that elicited reductions in IL‐6 protein (reported above) were further investigated for their effects on IL‐6 mRNA levels using RT‐qPCR following incubation with treatment compounds at their highest tested concentration (10 μM), for 24 h. IL‐6 mRNA expression was increased by 3.5 ± 0.9‐fold (*p* < 0.05, Fig. 3) in cells treated with oxLDL alone (oxLDL control) compared to un‐stimulated cells. All compounds except ferulic acid reduced oxLDL‐induced IL‐6 mRNA expression by ≥ 55.2 ± 7.3% of the oxLDL control (*p* ≤ 0.006, Fig. 3). VA exhibited the greatest reduction in IL‐6 mRNA levels, displaying an 82.9 ± 10.4% reduction relative to the control (*p* ≤ 0.001).

### 3.3 CD40L‐stimulated IL‐6 and VCAM‐1 production

**Figure 4:**
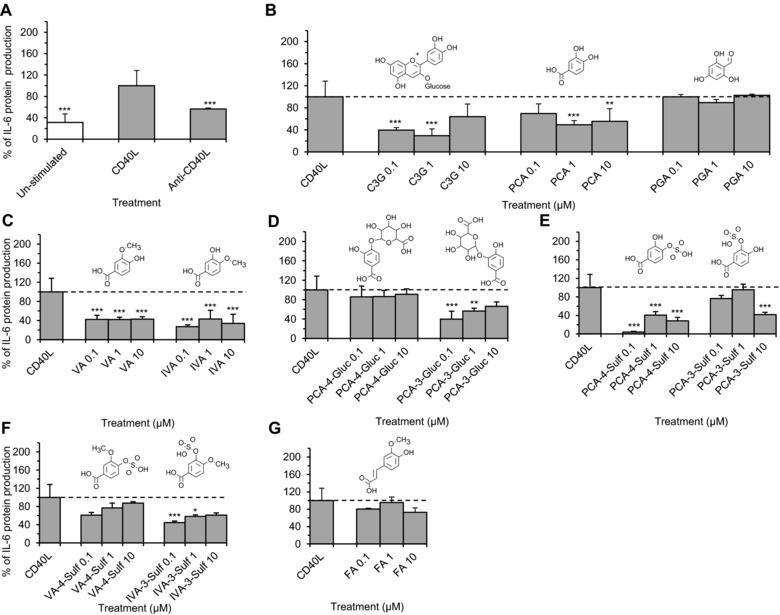
CD40L‐stimulated IL‐6 protein production in HUVECs co‐incubated with C3G, phenolic metabolites or CD40L controls for 24 h. (A) Cells incubated with or without D1.1 cells (1 × 10^6^ cells/well); (B–G) cells incubated with C3G or phenolic metabolites at 0.1, 1, 10 μM and D1.1 cells (1 × 10^6^ cells/well). All data expressed as mean percentage (± SD, *n* = 3) of CD40L‐induced controls. ***p ≤ 0.001, **p ≤ 0.01, *p ≤ 0.05 (ANOVA with Tukey post‐hoc) relative to CD40L‐stimulated control. C3G, cyanidin‐3‐glucoside; PCA, protocatechuic acid; PGA, phloroglucinaldehyde; VA, vanillic acid; IVA, isovanillic acid; PCA‐4‐Gluc, PCA‐4‐glucuronide; PCA‐3‐Gluc, PCA‐3‐glucuronide; PCA‐3‐Sulf, PCA‐3‐sulfate; PCA‐4‐Sulf, PCA‐4‐sulfate; VA‐4‐Sulf, VA‐4‐sulfate; IVA‐3‐Sulf, IVA‐3‐sulfate; FA, ferulic acid.

IL‐6 protein expression was significantly increased (3.2 ± 1.5‐fold) in CD40L‐treated HUVECs but reduced (44.4 ± 1.7%) when D1.1 cells were pre‐incubated with anti‐CD40L antibody, suggesting a direct role of CD40L in the production of IL‐6 in the present model (Fig. 4A). The parent anthocyanin and seven metabolites reduced CD40L‐induced IL‐6 protein production by ≥41.7 ± 3.5%, relative to cells treated with CD40L alone (CD40L control) (*p* ≤ 0.03, Fig. 4). C3G and PCA both reduced IL‐6 protein production by ≥44.6 ± 23.0% of the control (*p* < 0.05, Fig. 4B), while there was no effect of PGA. The conjugated metabolites of PCA, VA, IVA, PCA‐3‐glucuronide, PCA‐3‐sulfate, PCA‐4‐sulfate, and IVA‐3‐sulfate, reduced CD40L induced IL‐6 production by ≥41.7 ± 3.5% (*p*≤0.03) for at least one of the concentrations tested (Fig. 4C–F), with a maximum reduction of 95.8 ± 1.3% (*p* ≤ 0.001) observed for the sulfate conjugate of PCA, PCA‐4‐sulfate (Fig. 4E).

**Figure 5:**
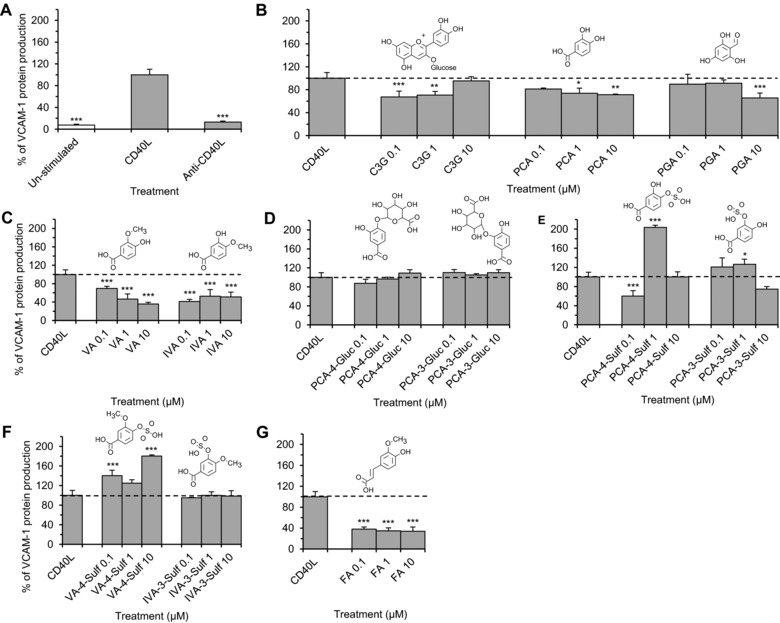
CD40L‐stimulated VCAM‐1 protein production in HUVECs co‐incubated with C3G, phenolic metabolites or CD40L controls for 24 h. (A) Cells incubated with or without D1.1 cells (1 × 10^6^ cells/well); (B–G) cells incubated with C3G or phenolic metabolites at 0.1, 1, 10 μM and D1.1 cells (1 × 10^6^ cells/well). All data expressed as mean percentage (± SD, *n* = 3) of CD40L‐induced controls. ***p ≤ 0.001, **p ≤ 0.01, *p ≤ 0.05 (ANOVA with Tukey post‐hoc) relative to CD40L‐stimulated control. C3G, cyanidin‐3‐glucoside; PCA, protocatechuic acid; PGA, phloroglucinaldehyde; VA, vanillic acid; IVA, isovanillic acid; PCA‐4‐Gluc, PCA‐4‐glucuronide; PCA‐3‐Gluc, PCA‐3‐glucuronide; PCA‐3‐Sulf, PCA‐3‐sulfate; PCA‐4‐Sulf, PCA‐4‐sulfate; VA‐4‐Sulf, VA‐4‐sulfate; IVA‐3‐Sulf, IVA‐3‐sulfate; FA, ferulic acid.

Co‐incubation of CD40L expressing D1.1 cells with HUVECs resulted in a 13.5 ± 1.3‐fold increase in VCAM‐1 protein production compared to un‐stimulated HUVECs (*p* ≤ 0.001, Fig. 5A). This effect was significantly reduced by 11.8 ± 0.4‐fold when D1.1 cells were pre‐incubated with anti‐CD40L antibody. The parent anthocyanin and six metabolites reduced CD40L‐induced VCAM‐1 protein production by ≥ 26.1 ± 8.8% (*p* ≤ 0.05) relative to the cells treated with CD40L alone (CD40L control). C3G, PCA and PGA reduced CD40L‐induced VCAM‐1 by ≤26.1 ± 8.8% (*p* ≤ 0.05; Fig. 5B), while the conjugated metabolites of PCA, VA, IVA, PCA‐4‐sulfate and ferulic acid, were more active, reducing VCAM‐1 production by ≥30.3 ± 4.5% (*p* ≤ 0.04), at one or more of the concentrations tested (Fig. 5C, E and G). The maximum reduction was observed for ferulic acid (65.9 ± 8.1%; *p* ≤ 0.001, Fig. 5G). An increase in VCAM‐1 protein production was observed when CD40L‐induced HUVECs were co‐incubated with PCA‐4‐sulfate (203.5 ± 4.5% at 1 μM, *p* ≤ 0.001), PCA‐3‐sulfate (126.5 ± 10.6% at 1 μM, *p* ≤ 0.04) and VA‐4‐sulfate (140.1 ± 10.8% at 0.1 μM and 180.3 ± 2.2% at 10 μM, *p* ≤ 0.001) relative to CD40L‐induced control (Fig. 5E and F).

### 3.4 CD40L‐stimulated IL‐6 and VCAM‐1 mRNA levels

**Figure 6:**
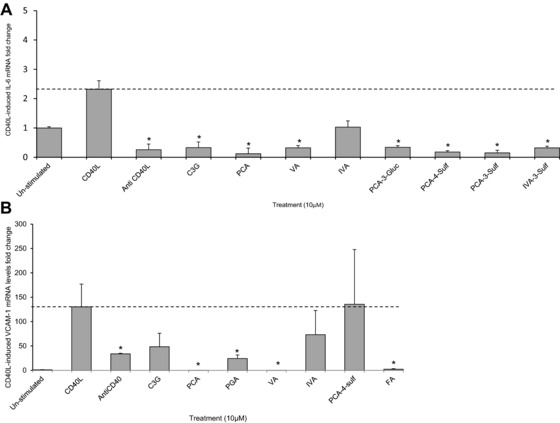
Change in CD40L‐stimulated IL‐6 and VCAM‐1 mRNA in HUVECs co‐incubated with C3G and phenolic metabolites or CD40L controls for 24 h. (A) IL‐6 mRNA fold change in cells co‐incubated C3G or phenolic metabolites (10 μM) with or without D1.1 cells (1 × 10^6^ cells/well). (B) VCAM‐1 mRNA fold change in cells co‐incubated with C3G or phenolic metabolites (10 μM) with or without D1.1 cells (1 × 10^6^ cells/well). All data graphically expressed as mean fold change (± SD, *n* = 3) relative to un‐stimulated (basal) IL‐6 and VCAM‐1 mRNA levels. *p ≤ 0.05 (ANOVA with Tukey post‐hoc) relative to CD40L‐stimulated control. C3G, cyanidin‐3‐glucoside; PCA, protocatechuic acid; PGA, phloroglucinaldehyde; VA, vanillic acid; IVA, isovanillic acid; PCA‐3‐Sulf, PCA‐3‐sulfate; IVA‐3‐Sulf, IVA‐3‐sulfate; FA, ferulic acid.

The parent anthocyanin and seven metabolites that reduced CD40L‐induced IL‐6 production as reported above, were further investigated for their effects on IL‐6 mRNA levels using RT‐qPCR following incubations with the treatment compounds at 10 μM for 24 h. CD40L stimulation increased IL‐6 mRNA expression in HUVECs by 2.3 ± 0.3‐fold (*p* ≤ 0.05, Fig. 6A), while anti‐CD40L reduced this below the levels observed in untreated HUVECs (0.2 ± 0.1‐fold, *p* ≤ 0.05). With the exception of IVA, all compounds reduced the levels of CD40L‐induced IL‐6 mRNA by ≥85.3 ± 2.5% (*p* ≤ 0.01, Fig. 6A) relative to the CD40L‐treated control. PCA caused the greatest reduction in CD40L‐induced IL‐6 mRNA expression, where IL‐6 mRNA levels were reduced by 94.9 ± 8.3% of the CD40L‐induced control (*p* ≤ 0.001).

The compounds which decreased CD40L‐induced VCAM‐1 protein concentrations (reported above) were investigated for their effects on CD40L‐induced VCAM‐1 mRNA levels, where VCAM‐1 mRNA was increased by 130.2 ± 46.1‐fold relative to untreated cells (*p* < 0.05, *n* = 3, Fig. 6B) and reduced by 96.4 ± 1.2‐fold when D1.1 cells pre‐incubated with anti‐CD40L antibody (*p* ≤ 0.05). The parent anthocyanin did not significantly reduce VCAM‐1 mRNA levels while four of the six metabolites tested reduced VCAM‐1 mRNA by ≥81.4 ± 7.2 (*p* ≤0.05, Fig. 6B). The greatest reduction in VCAM‐1 mRNA levels was observed for VA, which reduced CD40L‐induced VCAM‐1 by ≥99.9 ± 0.01% of the control (*p* ≤ 0.04).

## 4 Discussion

Epidemiological studies suggest greater levels of anthocyanin consumption are associated with a reduction in CVD risk 1, however, the mechanisms behind this association remain elusive. Most previous animal and in vitro studies have focused on the activity of the parent (un‐metabolized) anthocyanins in models of vascular function 5, 6, 7. The present study explored the activity of metabolites of anthocyanins which we recently identified in a stable isotope‐labelled anthocyanin intervention in humans 18. To the best of our knowledge this is the first in vitro study to examine the relative bioactivity of recently identified metabolites relative to their parent anthocyanin, using pure synthetic phase II conjugates at physiologically relevant concentrations. In the present study, we screened anthocyanin metabolites across a dose range of 0.1–10 μM, as it represents the relative concentration range observed following the consumption of anthocyanins in two recent studies in our lab 24, 25. Here, phenolic metabolites were reported ranging from 0.1–2 μM, with cumulative concentrations of metabolites reaching 10 μM. It should be noted that there was considerable variation in metabolite concentrations between individuals.

As anthocyanins have been shown to reduce oxLDL‐induced apoptosis 26 and dampen the effect of CD40‐CD40L interaction in endothelial cells 14, the activity of anthocyanin metabolites was explored using oxLDL‐ and CD40L‐stimulated endothelial cells. Here, we found that metabolites of C3G induced greater reductions in IL‐6 and VCAM‐1 protein compared to their parent structure, under both oxLDL and CD40L stimulation. This finding suggests that the degradation and subsequent metabolism of anthocyanins does not reduce their bioactivity and may in fact increase it.

Oxidized LDL is a potent chronic inflammatory activator and an established risk factor for CVD 27. OxLDL has also been shown to increase the levels of IL‐6 in vascular endothelial cells 28, however, very little is currently known of the effects of physiologically relevant anthocyanin metabolites on oxLDL‐mediated inflammation. As oxLDL is known to be proapoptotic at high concentrations (such as 100 μg/mL) 29, the present study utilized considerably lower concentrations (5 μg/mL) than many previous investigations 28 in an attempt to create a more physiologically relevant model of vascular dysfunction. The levels utilized in the present investigation are comparable to levels identified in the blood of patients with coronary artery disease 27. The present study is the first to investigate the effects of C3G metabolites on oxLDL‐induced IL‐6 production in vitro, and our findings suggest that the anti‐inflammatory effect of anthocyanins are not limited to the parent structures themselves, as their lower molecular weight phenolic metabolites displayed even greater bioactivity, as observed by reductions in oxLDL‐induced IL‐6 production by as much as 99% of control cells (Fig. 2E). This suggests that degradation and subsequent metabolic conjugation does not reduce the biological activity of anthocyanins but in some instances enhances it.

The activation of CD40 via its ligand, CD40L, is also associated with promoting chronic inflammation and the expression of pro‐inflammatory mediators, including IL‐6 14 and VCAM‐1 30 and therefore plays a pivotal role in the development of CVD. Very little is currently known of the effect of C3G metabolites on CD40L‐mediated inflammation. We observed extensive bioactivity of the phenolic metabolites relative to the parent anthocyanin, as observed by a reduction in CD40L‐induced IL‐6 and VCAM‐1 by as much as 95 and 65%, respectively (Figs 4E and 5G). The effects of C3G on CD40L‐induced IL‐6 and VCAM‐1 have previously been reported, where Xia et al. 14, 31 observed a significant reduction in CD40L‐induced IL‐6 and VCAM‐1 following incubation with C3G. Our findings are in accordance with this previous work and provide additional evidence for the activity of C3G metabolites.

In oxLDL‐stimulated HUVECs, the parent C3G had no effect on IL‐6 production, while many of the metabolites tested significantly decreased IL‐6 (Fig. 2). Amongst these, one of the degradation products, PCA, reduced oxLDL‐induced IL‐6 significantly, whereas PGA had no effect (Fig. 2B); suggesting the degradation of C3G to PCA may increase the bioactivity of the parent anthocyanin. All PCA conjugates tested, except IVA, reduced oxLDL‐induced IL‐6 significantly (32–99% reduction, Fig. 2C–G), suggesting further metabolism through methylation, glucuronidation or sulfation does not result in decreased bioactivity. Sulfate conjugation reduced oxLDL‐induced IL‐6 production to the greatest extent, compared to other conjugation reactions (methylation or glucuronidation). Sulfate conjugates reduced IL‐6 production by as much as 99% of control levels, suggesting sulfation of PCA has the greatest impact on anthocyanin bioactivity in the present model. The bioactivity of VA has previously been reported in, in vitro models by Kim et al. 20, where VA reduced IL‐6 production in LPS‐induced mouse peritoneal macrophages. Furthermore, in the present study VA reduced IL‐6 and VCAM‐1, under both stimulated conditions (oxLDL and CD40L), suggesting significant anti‐inflammatory activity. With regards to the other phase II conjugated derivatives of PCA tested (PCA‐3‐glucuronide, PCA‐4‐glucuronide, PCA‐3‐sulfate, PCA‐4‐sulfate, VA‐4‐sulfate and IVA‐3‐sulfate), this is the first in vitro study to report their anti‐inflammatory activity and provides evidence that metabolites of anthocyanins may contribute to reducing CVD risk by altering expression of inflammatory mediators.

In CD40L‐stimulated HUVECs, C3G and 7 metabolites reduced IL‐6 protein production (between 41 and 96% of CD40L control) at one or more concentration tested. C3G and its B‐ring degradation product, PCA, significantly reduced IL‐6 production (Fig. 4B), whilst PGA did not, again suggesting the catechol group imparts some anti‐inflammatory activity under CD40L‐stimulated conditions. However, in contrast to the oxLDL stimulated cell model, further metabolic conjugation of the catechol group does not appear to significantly reduce this activity. Of the conjugated metabolites of PCA tested, all compounds except PCA‐4‐glucuronide, VA‐4‐sulfate and ferulic acid, reduced CD40L‐induced IL‐6 production, with a maximum reduction observed for PCA‐4‐sulfate (95.8% reduction relative to CD40L control), suggesting sulfate conjugation has the greatest impact on anthocyanin bioactivity under the present CD40L‐stimulated conditions.

In CD40L‐stimulated HUVECs, C3G and 6 metabolites significantly reduced VCAM‐1 production (Fig. 5), where C3G and both its degradation products, PCA and PGA, reduced CD40L‐induced VCAM‐1 (Fig. 5B). Of the conjugates of PCA tested, ferulic acid had the greatest effect (Fig. 5G). The effects of sulfate and glucuronide conjugation were less apparent (Fig. 5D and E), which is in contrast to the effects observed for CD40L‐induced IL‐6, where sulfate conjugated PCA resulted in the greatest reductions in IL6 production. The findings from the present study provide support that anthocyanin metabolites possess anti‐inflammatory activity and the conjugation of the catechol group on the phenolic metabolites often leads to increased bioactivity. This differential bioactivity between parent and metabolite has also been reported for conjugated forms of other flavonoids, such as methylated (‐)‐epicatechin and sulfated and glucuronidated quercetin 32, 33, 34.

To the best of our knowledge, this is the first in vitro study to examine the effects of metabolites of C3G on IL‐6 and VCAM‐1 in CD40L‐stimulated endothelial cells. Previous studies using differing in vitro models have reported the anti‐inflammatory activity of PCA. For example, significant reductions in VCAM‐1 protein production have been reported 15, 35 in LPS–induced RAW264.7 and TNF‐α‐induced bovine aortic endothelial cells co‐incubated with PCA; which is in line with the present study where PCA reduced CD40L‐induced VCAM‐1 by greater than 26% of controls. The effects of PCA on TNF‐α‐induced VCAM‐1 has also been observed previously 15, although at much higher concentrations (≥20 μM) than utilized in the present study; where we observed effects on VCAM‐1 protein production at concentrations as low as 1 μM. Metabolite concentrations are reported to reach 1–2 μM in the plasma of human volunteers 18, which is in line with the concentrations utilized in the present investigation and below those utilized in many previous studies. Moreover, in a ^13^C‐labelled C3G bioavailability study in humans, Czank et al. reported 0.14 μM of C3G in 2 h serum samples 18, and C3G was bioactive at this concentration under CD40L stimulated conditions in the present investigation, suggesting that C3G may also contribute to the anti‐inflammatory effect in vascular endothelium; albeit for a limited time post consumption.

It is worth noting that we observed differential effects under different stimulus for several of the metabolites studied. For example, IVA reduced IL‐6 protein production significantly in CD40L‐stimulated HUVECs (by 56–73% of CD40L control) but had no effect on oxLDL‐challenged HUVECs (Figs. 2C and 4C). Similarly, ferulic acid significantly reduced oxLDL‐induced IL‐6 production (by 32–42% of oxLDL control) but did not alter CD40L‐stimulated IL‐6 production (Figs. 2G and 4G). Together these data indicate that the anti‐inflammatory activity of anthocyanin metabolites differs depending on the inflammatory stimulus present, suggesting that these compounds may act by targeting unique signalling pathways (i.e., unique to either CD40L or oxLDL signaling) potentially through acting on tumor necrosis factor alpha receptor associated factor‐2 (TRAF‐2) 14 during CD40L signaling and LOX‐1 (oxLDL receptor) during oxLDL signalling 36. As such, this ability to influence multiple pathways may ultimately result in additive effects on CVD risk; however, it is clear that further research is necessary to establish the direct pathways affected.

In most cases, metabolites that reduced protein levels of IL‐6 and VCAM‐1 also reduced IL‐6 and VCAM‐1 mRNA levels. In oxLDL‐stimulated HUVECs, all bioactive compounds, except ferulic acid, reduced IL‐6 mRNA levels between 55 and 83% of controls. Similarly, in CD40L‐stimulated HUVECs, all compounds except IVA reduced mRNA levels of IL‐6 (between 85 and 95% of the CD40L control); indicating that these metabolites alter IL‐6 protein production by inhibiting CD40L‐induced IL‐6 mRNA induction. CD40L‐induced VCAM‐1 mRNA levels were also reduced by PCA, PGA, VA and ferulic acid, indicating bioactivity at the translational level. While this evidence may explain the link between gene expression and protein level, in several instances the correlation between protein (Figs. 2, 4 and 5) and mRNA (Figs. 3 and 6) was not apparent. Such divergence may be the result of post translational modification (PTM) of IL‐6 and VCAM‐1, as IL‐6 is subject to phosphorylation and glycosylation 37, 38, 39 and a proteolytic cleavage is required to release membrane bound VCAM‐1 to produce soluble VCAM‐1 40.

The present study was an initial step in understanding the bioactivity of anthocyanin metabolites. The primary goal of the present study was to investigate whether the metabolism of anthocyanins results in increased or decreased activity relative to the parent unmetabolised structure. A possible mechanism by which metabolites may regulate mRNA levels of IL‐6 and VCAM‐1 is by modulating the activation of NF‐κB, as IL‐6 and VCAM‐1 are regulated by NF‐κB. Anthocyanin‐enriched extracts have been shown to inhibit the activation of NF‐κB in, in vitro studies, where blueberries 17 and black rice extract 35 reduced NF‐κB activation. Furthermore, PCA, VA and ferulic acid, in various in vitro models have been demonstrated to inhibit NF‐κB activation 19, 20, 41. C3G has also been reported to attenuate NF‐κB through impairing the translocation of TRAF‐2 to lipid rafts in endothelial cells 14. Lastly, the reduced size and altered solubility of the metabolites could influence membrane penetration and increase access to intracellular mechanisms 42, 43

The present study examined the effect of anthocyanin metabolites at three dietary relevant concentrations (0.1, 1 and 10 μM), where the dose response appeared non‐linear except in the case of VA, which appeared to reduce CD40L‐induced VCAM‐1 production in a linear dose‐dependent manner (Fig. 5C). This observed non‐linear dose response for the other treatments has previously been reported for other flavonoids such as quercetin and genistein and may have important biological implications 44, 45.

While the present study provides novel insights into the anti‐inflammatory activity of recently identified anthocyanin metabolites, there are several limitations with this experimental approach. First, the measurement of IL‐6 and VCAM‐1 protein under stimulated conditions was carried out at a single time point (at 24 h stimulation) and the effects may differ following shorter periods of incubation 46, 47. Further time‐course studies are required to establish time‐dependent effects. The present study also used a co‐incubation model of activity and effects may differ following pre‐ or post‐stimulation (CD40L or oxLDL) with anthocyanin metabolites. Additionally, the investigation of the effect of bioactive metabolites on mRNA levels of IL‐6 and VCAM‐1 was performed only at the maximal treatment concentration utilised in the screen (i.e., 10 μM). The highest dose in the screen was chosen to demonstrate maximal activity at the translational level, however responses may have differed at 0.1 or 1 μM. Future work should explore a greater range of physiologically appropriate concentrations. This initial screen of anthocyanin metabolite activity was designed to establish if the metabolism of anthocyanins altered their anti‐inflammatory activity; and here we explored activity on only a limited number of chemokines (IL‐6 and VCAM1), however an extensive array of biologically important chemokines, such as MCP‐1, ICAM‐1, IL‐12, IL‐10, IL‐1β (to name a few) require future study.

The present study used D1.1 cells as a primary source of CD40L. D1.1 (Jurkat) cells are T‐cells that express high levels of CD40L and are much more cost effective than using recombinant CD40L for large screening experiments. This could also be considered a limitation of the present work as the D1.1 cells add a level of ambiguity to the model system. However, as our primary aim was to determine if metabolism of anthocyanins alters their bioactivity, D1.1 cells provided a cost effective alternative to recombinant CD40L. In addition, the use of D1.1 cells as a primary source of CD40L to investigate inflammatory responses is a common method reported in the literature 48, 49, 50. However, the results should be confirmed in future studies using pure recombinant CD40L (soluble CD40L). In addition, even though HUVECs are a well‐established model for endothelial cell research, these results should be confirmed using in vivo studies. Finally, soluble VCAM‐1 was quantified in the current investigation as opposed to membrane bound VCAM. The use of membrane bound over soluble VCAM‐1 as a marker of CVD risk has been a current topic of discussion in the literature and Videm et al. has recently published a study summarizing the relationship between VCAM‐1 mRNA levels and concentration of membrane bound and soluble VCAM‐1 40. The authors concluded that there was no trend between mRNA levels and VCAM‐1 protein that would favour the selection of membrane bound VCAM‐1 over soluble VCAM‐1 as a biomarker of activity. The study concluded that soluble VCAM‐1 appeared to be a better biomarker than membrane bound VCAM‐1 in HUVECs. Future work could however verify these associations with membrane bound VCAM‐1 protein to establish if anthocyanin metabolites interfere with its proteolytic cleavage at the membrane, as previously suggested 40. This aside, the present study provides a screen for the biological activity of a wide range of previously unstudied anthocyanin metabolites and provides a clear future direction for anthocyanin research.

Our novel data highlight that C3G metabolites are bioactive at physiologically relevant concentrations. Further research is required to establish not only the underlying mechanisms involved but also the physiological relevance of these findings to humans. These studies provide the first evidence that anthocyanin metabolites possess anti‐inflammatory effects that are likely to contribute to the reduced risk of CVD associated with increased habitual intake of anthocyanins 1.

*All other authors have declared no conflict of interest*.
